# Long-term follow-up of linear scleroderma en coup de sabre in children with central nervous system involvement

**DOI:** 10.3389/fimmu.2026.1740848

**Published:** 2026-02-09

**Authors:** Xingzhi Chang, Lihong Ren, Ye Wu, Yuehua Zhang, Cuijie Wei, Qingping Zhang, Meijiao Zhang, Chunyan Zhao, Xinhua Bao

**Affiliations:** 1 Department of Pediatrics, Peking University First Hospital, Beijing, China; 2 Department of Pediatrics, Chengdu Women's and Children's Central Hospital, School of Medicine, University of Electronic Science and Technology of China, Chengdu, Sichuan, China

**Keywords:** central nervous system involvement, immunotherapy, linear scleroderma en coup de sabre, long-term follow-up, magnetic resonance imaging, pediatrics

## Abstract

**Introduction:**

Linear scleroderma en coup de sabre (ECDS) is a rare disorder that often involves the central nervous system (CNS), requiring systemic immunotherapy. This study characterizes the clinical and neuroimaging features as well as the long-term treatment outcomes of pediatric ECDS.

**Methods:**

Patients with ECDS and CNS involvement were enrolled. Clinical manifestations, cranial imaging, pathology, and immunotherapy responses were documented.

**Results:**

Seven patients (6 females and 1 male) were included, with onset ages ranging from 1.8 to 13.5 years. Rash preceded neurological symptoms in five patients; seizures were the initial manifestation in the remaining two. Seizures were the most common neurological symptom (5/7), followed by dizziness (3/7), movement disorder (2/7), blurred vision (1/7), and headache (1/7). All exhibited ipsilateral supratentorial MRI abnormalities, exclusively on the left side and predominantly frontal. White matter lesions were observed in all patients, and cyst-like lesions were identified in four. Brain biopsy performed in two patients indicated vasculitis. All received systemic corticosteroids, either alone (2 cases) or combined with other agents (methotrexate in 5, mycophenolate mofetil in 3, IVIg in 3, tocilizumab in 2, and rituximab in 1). Over 6 months to 15 years of follow-up, neurological symptoms resolved in five patients. Skin lesions progressed in three patients, stabilized in two, and improved in two.

**Conclusion:**

Linear scleroderma en coup de sabre with CNS involvement predominantly affects females and typically involves the left cerebral hemisphere. Characteristic brain MRI findings include ipsilateral supratentorial white matter abnormalities and cyst-like lesions. Combination therapy with systemic corticosteroids and methotrexate is recommended as first-line treatment, while tocilizumab may be beneficial for refractory cases.

## Introduction

Localized scleroderma (also named morphea) is a rare inflammatory disorder of the skin and subcutaneous tissue, characterized by collagen deposition and fibrosis of the skin and soft tissues (1). It is classified into four main subtypes: circumscribed morphea, generalized morphea, pansclerotic morphea and linear morphea. Linear scleroderma (LS) is the most common subtype of localized scleroderma and predominantly affects children (2). To date, LS remains a rare and poorly understood condition, with an reported incidence of 2.5 per million children per year in the UK and Ireland (3). LS typically presents with linear atrophy and/or hardening of the skin and subcutaneous tissue, occasionally extending to involve underlying muscles and bones (4). When LS involves the head region (such as the forehead, scalp, or chin), it is often termed “en coup de sabre” (ECDS), due to the resemblance of the skin lesions to the stroke of a sabre (5). Whether progressive hemifacial atrophy (PHA), or Parry-Romberg syndrome represents a more severe form of localized craniofacial scleroderma remains a subject of debate (6). Extra-cutaneous involvements, including neurological abnormalities, ocular complications, and cosmetic morbidity, are rising and more frequently encountered in children than in adult-onset localized scleroderma (7). Neurological involvement has been observed in 19% of children with localized craniofacial scleroderma (8), and it occurs almost exclusively in LS en coup de sabre (ECDS) (9, 10). Common neurological manifestations include seizures and headaches, although other symptoms such as focal neurological deficits may also occur (11, 12).

Treatment of LS depends on its subtypes, the depth of tissue involvement, and disease activity. The European Dermatology Forum (EDF) experts recommend topical agents and ultraviolet phototherapy for LS limited to the skin, and methotrexate (either as monotherapy or in combination with systemic corticosteroids) for LS ECDS (1, 12). However, most reported LS therapies have focused primarily on skin manifestations. In a systematic review investigating the treatment of LS ECDS, which included 34 articles (4 retrospective cohort studies, 2 prospective cohort studies, 4 case series, and 24 case reports), only 3 out of 69 patients received treatment for extracutaneous (neurological and ophthalmic) involvement (5). The clinical course and long-term outcomes of LS with CNS involvement remain poorly understood, as only 15 cases with detailed treatment response data have been identified in the literature (**Supplementary Table S1**). Herein, we present the clinical and neuroimaging characteristics, as well as the long-term outcomes of immunotherapy, with a focus on the treatment of CNS involvement in seven pediatric patients with LS.

## Methods

We reviewed the medical records of pediatric patients (<18 years) diagnosed with “Linear scleroderma en coup de sabre” who visited the Pediatric Neurology Department of our hospital between June 1, 2004, and June 30, 2022. Seven patients meeting all the following criteria were included: (1) presence of en coup de sabre (2) presence of newly developed neurological symptoms; and (3) completion of serial cranial MRI scans (at least two examinations). Detailed clinical data was collected included demographics, age at onset and diagnosis, clinical manifestations of cutaneous and neurological systems, results of serological and cerebrospinal fluid tests, pathological features of skin or brain biopsy (if performed), immunotherapy regimens (drugs, doses, and duration), and treatment response. All cranial MRI scans were reviewed by a neuroradiologist masked to the clinical outcomes. Follow-up imaging was performed to assess for resolution or progression of imaging abnormalities at 6–12 months intervals and when there were acute neurological manifestations. Treatment response was categorized as improved, stable, or deteriorated in three domains: skin lesions, CNS symptoms, and cranial MRI findings. Improvement was defined as absence or reduction in the extent of skin lesion, substantial resolution of neurological symptoms, and disappearance or reduction of active lesions on follow-up MRI. Stability was defined as no significant changes in the extent of skin lesions, CNS symptoms, or MRI abnormalities. Deterioration was defined as enlargement of pre-existing skin lesions or development of new skin lesions, recurrence or worsening of CNS symptoms, or enlargement of pre-existing MRI lesions or emergence of new lesions. All patients were followed up at the outpatient clinic for 6 months to 15 years (4.11 ± 4.88 years).

## Results

### Clinical manifestations

A total of seven patients (six females, one male) diagnosed with LS ECDS were enrolled (**Table 1**). The age at onset ranged from 1.8 to 13.5 years (7.3 ± 3.5 years). All patients presented with skin abnormalities involving the left forehead and/or scalp. Five patients had a skin rash as the initial symptom, which initially manifested as linear erythema or a furuncle before progressing to a typical “knife-cut” depression. In the remaining two patients (Cases 2 and 5), the onset time of the rash was unknown, as their initial symptom was seizures. A “knife-cut” depression on the left forehead was noticed at their first visit to our institution.

**Table 1:** Clinical and imaging characteristics of patients with linear scleroderma en coup de sabre.

Case	Sex	Age at onset (y)	Initial symptom	Age at diagnosis (y)	Neurological symptoms	MRI abnormalities	MRI lesion location (side/lobe)	Contrast enhancement on MRI	Pathology(site/findings)
1	F	6	Rash	8	Seizure	Multiple WMAS, CL	Left/frontal, temporal	+	Brain/vasculitis, demyelination
2	M	13.5	Seizure	13.6	Dizziness, seizure	Multiple WMAS	Left/frontal, corpus callosum	–	N/A
3	F	8.5	Rash	9.8	Seizure	Multiple WMAS, CL	Left/frontal, temporal	–	Skin/collagen deposition, vasculitis
4	F	6	Rash	8.5	Dizziness, blurred vision	Multiple WMAS,	Left/frontal, temporal, occipital, corpus callosum	+	N/A
5	F	7	Seizure	7.8	Seizure headache, dyskinesia	WMAS, CL	Left/frontal	+	Brain/vasculitis, calcification, hemosiderin deposition, malacia foci
6	F	8.3	Rash	8.5	Dizziness, movement disorder	Multiple WMAS,	Left/frontal, putamen, insula	–	N/A
7	F	1.8	Rash	1.9	Seizure	Multiple WMAS, CL	Left/frontal, corpus callosum	+	Skin/collagen deposition, vasculitis

CL, cyst-like lesion; F, female; M, male; m, month; N/A, data unavailable; WMAS, white matter abnormal signal; y, year; +, meningeal and white matter enhancement; -, no enhancement.

All patients developed symptoms of CNS involvement. The interval between rash onset and the development of neurological manifestations ranged from 1 month to 2.5 years (except two patients with unknown onset time of rash), and the age at onset of neurological symptoms ranged from 1.9 to 13.5 years. Focal seizures were the most common neurological symptom (5/7 patients). Other neurological symptoms included dizziness (Cases 2, 4, and 6), movement disorder (Cases 5, 6), blurred vision (Case 4) and headache (Case 5). For the two patients without seizures: one (Case 4) experienced paroxysmal dizziness, vomiting, and blurred vision; the other (Case 6) presented with transient dyskinesia, dysarthria, and dizziness.

### Neuroimaging characteristics

All patients underwent repeated cranial MRI examinations (**Table 1**). MRI abnormalities were detected prior to the onset of neurological symptoms in one patient (Case 1), concurrently with neurological symptom presentation in three patients (Cases 2, 6 and 7), and after appearance of neurological symptoms in three cases (Cases 3, 4 and 5). In Case 1, cranial MRI was performed due to scalp atrophy, subsidence and hair loss on the left forehead. Abnormal white matter signals in the left centrum semiovale were identified 2 years before neurological symptoms developed. In Cases 2 and 7, abnormal signals in the left frontal lobe, hypothalamus, and corpus callosum were detected on the same day that neurological symptoms appeared, whereas in Case 6, these were observed 7 days after symptom presentation. Regarding Cases 3, 4 and 5, MRI abnormalities were detected 2 months, 1 year, and 3 months after the onset of neurological symptoms, respectively.

All patients exhibited white matter signal abnormalities. Cyst-like lesions were observed in four patients: at the initial MRI examination in Cases 3, 5 and 7, and during an MRI follow-up examination 2 years later in case1. In Case 5, the cyst-like lesion was initially mistaken for a tumor due to an accompanying mass effect. Leptomeningeal contrast enhancement was detected in four patients (Cases 1, 4, 5 and 7). Cranial computed tomography (CT) scans were conducted in six patients (all except Case 2), revealing multiple calcifications in five (excluding Case 1, who underwent CT scanning very early in the clinical course). Additionally, bleeding adjacent to the cyst-like lesion was identified in Case 5.

All MRI abnormalities were ipsilateral to the facial skin lesions. Regarding the anatomical distribution of these abnormalities, the most common locations were the frontal and parietal lobes, which were affected in all patients, followed by the temporal lobe in three cases (Cases 1, 3 and 4), the corpus callosum in three cases (Cases 2, 4 and 7), the occipital lobes in one case (Case 4), and the left putamen and insula in one case (Case 6). All lesions were localized to the supratentorial regions.

### Laboratory investigation

Routine blood tests, biochemistry investigations, and examinations for autoimmune antibodies (including antineutrophil cytoplasmic antibodies, antinuclear antibody, and rheumatoid factor) were normal. Cerebrospinal fluid (CSF) analysis was performed in six patients (excluding Case 3). CSF profiles were normal in three patients, while pleocytosis (50, 20, and 34 white blood cells/mm^3^) was noted in three patients (Cases 2, 6, and 7) who showed normal glucose and protein levels. CSF culture results were negative. Oligoclonal bands in CSF were positive in three cases (Cases 2, 6 and 7). Tests for autoimmune antibodies, including anti-N-methyl-D-aspartate receptor (anti-NMDAR), anti-myelin oligodendrocyte glycoprotein (anti-MOG), and anti-aquaporin-4 (anti-AQP4) antibodies, were negative in five patients with available data.

Skin biopsies were performed in Cases 3 and 7. Pathological examinations revealed collagen deposition and perivascular inflammatory cells infiltration. Brain biopsy was performed in Cases 1 and 5 after the onset of neurological symptoms, at 2 years and 3 months of clinical course, respectively. Pathological examination showed perivascular infiltration by inflammatory cells, thickening and calcification of the vascular wall, neuronal degeneration, and white matter demyelination.

### Treatment and prognosis

All patients received systemic immunosuppressive therapy. Specific drug names and treatment durations for each patient are detailed in **Table 2**. The dosage and administration of each medication were as follows: high-dose methylprednisolone (15–20 mg/kg/day for 3 days), followed by oral prednisone (1–1.5 mg/kg/day) with gradual tapering; methotrexate (10–15 mg/m²/week); cyclophosphamide (500–750 mg/m²/month); mycophenolate mofetil (800–1200 mg/m²/day); intravenous immunoglobulin (IVIg; 2 g/kg/month); rituximab (375 mg/m²/week for 2 consecutive weeks, then repeated at 375 mg/m² every 3–6 months when the B-lymphocyte percentage exceeded 0.1%); and tocilizumab (12 mg/kg/month).

**Table 2:** Treatment response of patients with linear scleroderma en coup de sabre.

Case	Sex	Immunosuppressive therapy (drugs/duration(m))	Follow-up (y)	Skin outcome	CNS outcome	MRI follow-up
1	F	CS/12	15	Deteriorated	Improved	Improved, deteriorated, finally stable
2	M	CS/6	0.6	Stable	Improved	Stable
3	F	CS/3, MTX/18	3	Stable	Improved	Improved
4	F	CS/4, MTX/12	2	Improved	Improved	Improved
5	F	CS/12, CTX/6, MMF/12	2.2	Deteriorated	Deteriorated	Deteriorated
6	F	CS/24, IVIg/3, MTX/6, Rituximab/12, MMF/18, Tocilizumab/15	3.5	Deteriorated	Improved	Initially improved, then deteriorated, finally stable
7	F	IVIg/3, CS/12, MTX/12, MMF/16, Tocilizumab/20	2.5	Improved	improved,	Initially improved, then deteriorated, finally stable

CNS, central nervous system; CS, corticosteroids; CTX, cyclophosphamide; F, female; IVIg, intravenous immunoglobulin; M, male; MMF, mycophenolate mofetil; MTX, methotrexate; m, month; y, year.

**Figure 1:**
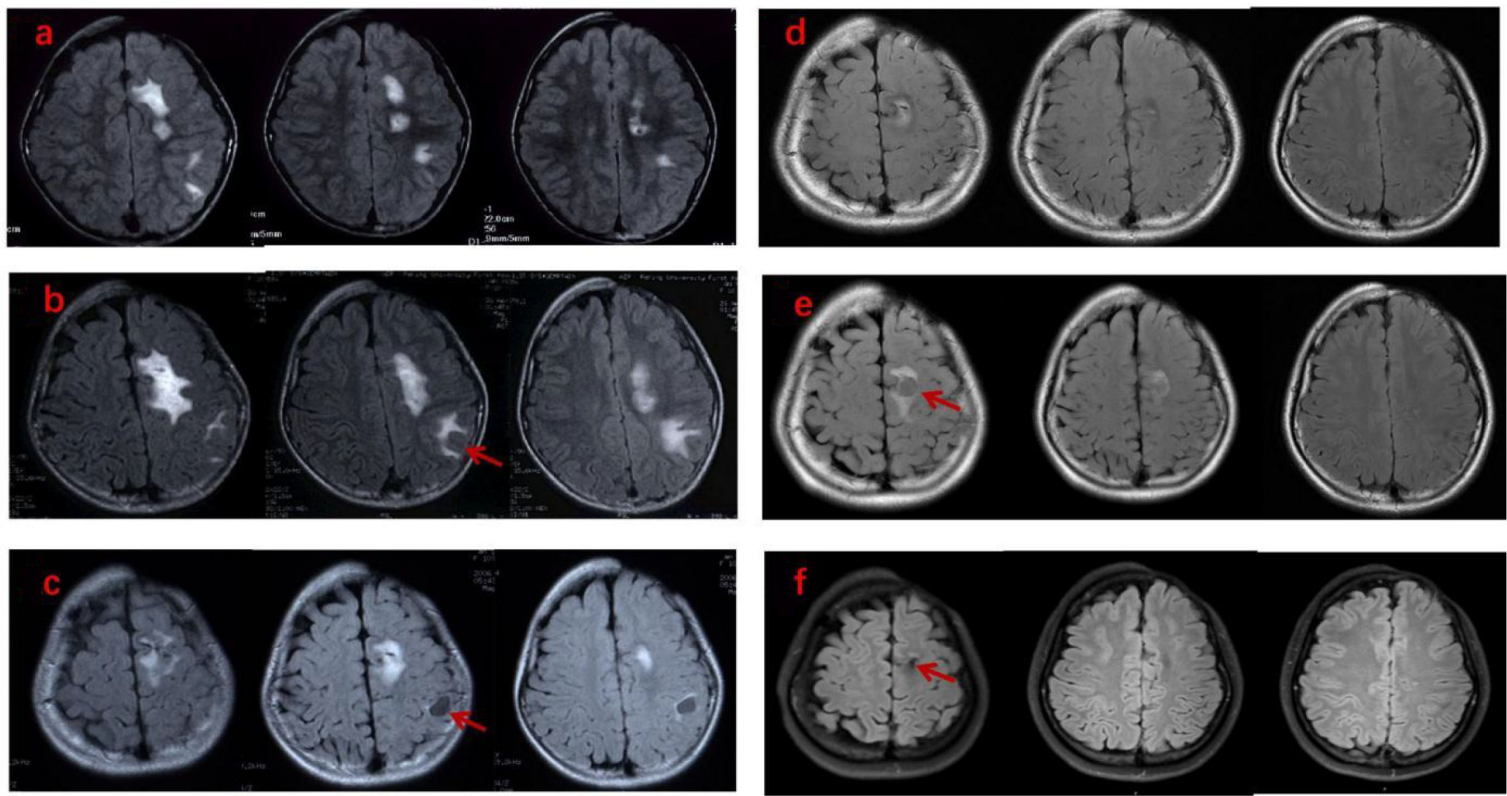
Evolution of neuroimaging findings in a patient with LS ECDS (Case 1). Axial T2-weighted fluid-attenuated inversion recovery (FLAIR) sequences showed multifocal hyperintensities in the left frontal and parietal lobes, which were ipsilateral to the facial skin lesions. Cyst-like lesions(arrowhead)appeared, resolved, and then reappeared in different locations, ultimately regressing during the 15-year follow-up period. **(a**, 2 years; **b**, 3 years; **c**, 4 years; **d** 8 years; **e**, 9 years; **f**, 15 years of follow-up**)**.

The response to treatment was evaluated across three domains (**Table 2**). ① Skin lesions remained stable or improved in four patients, but deteriorated in three, which was not parallel to the response of neurological symptoms. ② Seizure control was achieved shortly after treatment (within 3 months) and maintained seizure-free status in three cases (Cases 1, 2, and 3) during a follow-up period of up to 15 years; In Case 7, seizure frequency fluctuated during treatment but ultimately remained seizure-free for 8 months over a 2.5-year follow-up. In Case 5, 1 to 3 daily seizures persisted, accompanied by persistent right limb dyskinesia. In Cases 4 and 6 (without a history of seizures), neurological symptoms resolved within 1 month after the initiation of treatment, and did not recur during follow-up. ③Brain lesions improved or stabilized in 6 of 7 patients at their last follow-up. Three evolutionary patterns were observed:ⓐ MRI abnormalities stabilized or improved and remained stable during follow-up in three cases (Cases 2, 3 and 4), accompanied by the resolution of neurological symptoms. ⓑMRI abnormalities fluctuated without neurological symptoms in two cases (Cases 1 and 6), initially improving, then exacerbating, stabilizing, deteriorating, and finally stabilizing. In Case 1, these lesions varied spontaneously without additional immunosuppressive therapy (**Figure 1**); in Case 6, intensive combined immunosuppressive therapy was subsequently administered when brain lesions progressed. ⓒBrain lesions progressed in parallel with neurological symptoms in two cases (Cases 5 and 7).Serial brain MRI showed transient improvement with reduced seizure frequency at the 6-month follow-up, followed by subsequent deterioration along with persistent seizures. Further intensive immunosuppressive therapy was then administered. In Case 7, brain abnormalities ultimately improved and remained stable thereafter; in Case 5, however, brain abnormalities progressed slowly with persistent daily seizures.

## Discussion

Central nervous system involvement in linear scleroderma is extremely rare. Only case reports and small case series have been reported (13). Neurological involvement typically develops after cutaneous manifestations (12); occasionally it may occur prior to or synchronously with cutaneous findings (9, 14, 15). In young children who first present with neurological symptoms, LS could be easily missed in the early stages (16). In the present study, two of the seven cases (Cases 2 and 5) presented with seizures as the initial symptom; the diagnosis was not confirmed until skin rashes were recognized 1 and 9 months later, respectively. For patients with typical dermatological changes, early and serial brain MRI is crucial to identify CNS involvement, especially in asymptomatic individuals. In Chiu’s report, 19% of children with ECDS or PRS showed intracranial abnormalities on MRI, half of whom were asymptomatic (17). All patients in this cohort exhibited both neurological symptoms and abnormal MRI findings. Notably, the interval between the onset of skin rash and neurological symptoms can be as long as 2.5 years, which emphasizes the importance of serial MRI examinations.

In patients with ECDS, MRI abnormalities are usually located ipsilateral to extracranial lesions, most of which are restricted to the supratentorial regions (11, 18). Both left-side (19, 20) and right-side (15) involvement have been reported in the literature, with a predominance on the left side (10, 18, 21). White matter abnormalities in the frontal lobe are the most common MRI findings, followed by those in the parietal, temporal, and occipital lobes (21). Consistent with previous reports, all seven patients in this cohort had left-sided white matter abnormalities, which were ipsilateral to their skin lesions. In contrast to previous reports, cyst-like lesions were more common in our cohort (4/7). Brain biopsy in Case 1 revealed white matter demyelination and perivascular inflammation, suggesting that vasculitis may contribute to the formation of these cyst-like lesions. This is consistent with a previous case report (22)and other histopathological studies of brain involvement in LS ECDS (23–25).

Management of LS varies in the literature, and optimal therapy remains unknown. It has been reported that juvenile-onset LS ECDS can exhibited persistent disease activity in adulthood (26). Systemic treatment is commonly employed for active LS with CNS involvement in most case series (10) and case reports (27). MTX combined with corticosteroids is the first-line treatment according to the consensus of the European Society of Pediatric Rheumatology (28), which is also our practice principle. All our patients received corticosteroids, with five of them combined with MTX. With respect to other immunosuppressants, mycophenolate mofetil is considered as a second-line treatment in current United Kingdom clinical practice (29), additionally, tocilizumab (25) and cyclophosphamide (19) have been occasionally utilized in case reports, and these agents were also administered in our patients due to recurrence of neurological symptoms and/or progression of MRI abnormalities. Due to the small sample size, It was difficult to determine which agent was more effective. However, it is interesting to note that two cases (Cases 6 and 7) failed conventional immunosuppressive therapies but ultimately achieved significant improvement with tocilizumab. Together with previous reports of successful treatment with tocilizumab (25, 30) We suggest that tocilizumab may be a viable option for refractory LS cases.

Treatment responses vary across the literature. Improvements in MRI findings following immunosuppressive treatment have been reported (31),while MRI lesions progressed despite intensive immunosuppressive therapy have also been reported (32). There is no standard method to evaluate the response. The inconsistency between neurological symptoms and neuroimaging findings increases the complexity of evaluation. It has been reported that some patients with neurological symptoms had normal imaging findings (21), whereas some patients with brain imaging abnormalities were asymptomatic (17). A similar phenomenon was observed in this study. Brain lesions progressed with fluctuations without recurrence of neurological symptoms were observed in two cases (Cases 1 and 6). It is important to note that in Case 1, MRI abnormalities fluctuated and stabilized spontaneously in the absence of further immunosuppressive therapy, whereas intensive immunotherapy was applied due to progressive MRI abnormalities in Case 6. For children with ECDS, contrast-enhanced brain MRI was recommended at baseline and upon the development of any new neurological symptoms (28).However, the clinical significance of asymptomatic MRI changes is unclear, and it is unknown whether treatment should be modified based solely on MRI abnormalities. Given the side effects of immunosuppressant in children, we suggest that both neurological symptoms and MRI findings in asymptomatic patients with ECDS should be monitored closely and carefully evaluated, and the use of immunosuppressants should be considered with caution.

## Conclusion

LS en coup de sabre, a rare subtype of localized scleroderma, most frequently affects girls and may be accompanied by CNS involvement ipsilateral to the craniofacial skin lesions. In LS ECDS, supratentorial white matter lesions represent the most common neuroimaging abnormality, with cyst-like lesions also being frequently observed. Notably inconsistencies exist between skin lesions and CNS involvement, as well as between neurological symptoms and MRI abnormalities. Corticosteroids combined with MTX are regarded as the first-line therapy, while tocilizumab may be beneficial for refractory cases. To optimize treatment and improve prognosis, close long-term follow-up and repeated evaluation are essential for all patients with LS ECDS.

## Data Availability

The original contributions presented in the study are included in the article/**Supplementary Material**. Further inquiries can be directed to the corresponding author.
