# Supplementary material for: Long-term follow-up of linear scleroderma en coup de sabre in children with central nervous system involvement
Source: Front Immunol. 2026 Feb 9;17:1740848. doi: 10.3389/fimmu.2026.1740848 (PMC12926168; doi:10.3389/fimmu.2026.1740848)
Supplement: Supplementary file 1 [file DataSheet1.pdf]

Supplementary table1 Clinical characteristics and treatment response of pediatric patients with linear scleroderma en coup de sabre accompanied by CNS involvement (in literature)

**case**	**sex**	**Age at onset (y)**	**Initial symptom**	**Gap between skin and CNS Symptom(m)**	**CNS symptoms**	**MRI abnormalities**	**MRI lesion location (side/lobe)**	**Treatment (drugs)**	**Follow-up (y)**	**Skin outcome**	**CNS outcome**	**Article (PMID)**
1	F	9	Seizure	48	Seizure headache	White matter abnormalities	Right / frontal	CS, MTX	2	Deteriorated	Improved	25404727
2	F	8	Headache	12	Headache	N	N	CS	0.6	Stable	Improved	25404727
3	F	6	Headache	36	Headache	N	N	CS, MTX	3	Stable	Improved	25404727
4	F	13	Headache	6	Headache	N	N	CS, MTX	2	Improved	Improved	25404727
5	F	8	Seizure	9	Seizure headache, dyskinesia	Micro-bleeds	Left/frontal , parietal	CS, MTX	2.2	Improved , deteriorated, stable	Improved	30168188
6	F	2.5	Seizure	2	Seizure	White matter abnormalities	Left/frontal, putamen, insula	CS, MTX, Tocilizumab	3.5	Deteriorated	Improved	29929808
7	M	10.5	Seizure	18	Seizure, headache	White matter abnormalities cystic encephalomal	Left/frontal,basal ganglia	CS, MTX	2.5	Improved	Improved	29961525

case	sex	Age at onset (y)	Initial symptom	Gap between skin and CNS Symptom(m)	CNS symptoms	MRI abnormalities	MRI lesion location (side/lobe)	Treatment (drugs)	Follow-up (y)	Skin outcome	CNS outcome	Article (PMID)
						acia micro-hemorrhages						
8	F	3	Rash	2	Seizure	White matter abnormalities	Left/frontal	CS, MTX	3	improved	Improved	9457714
9	F	10	Seizure	0	Seizure	White matter abnormalities	Leftl/ frontal,basal ganglia	CS, MTX	0.5	Improved	Improved	26860318
10	M	6	Swollen eye	3	Headache, seizure	White matter abnormalities	Left/frontal	CS, MTX	0.5	Improved	Improved	24667737
11	F	6	Seizure	0	Seizure	White matter abnormalities	Right/frontal, parietal	CS, MTX, CTX	4	Improved, Deteriorated	Improved, then lost follow-up	24667737
12	F	9	Rash	48	Seizure, headache	White matter abnormalities	Left/parietal,occipital	CS, MTX, IVIg, CTX	2	Stable	Improved	31670009
13	M	6	Seizure	4	Seizure behavioural abnormalities	White matter abnormalities	Left/frontal	CS, MTX	3	Stable	Improved	19796565
14	F	2.8	Seizure	12	Seizure cognitive regression	Atrophy, calcification	Right/hippocampal	MTX	2	N/A	Stable	18207439
15	M	1.7	Seizure	12	Seizure	White matter abnormalities	Left /frontal,parietal	CS, MTX	10	Improved	Improved	33820899

Note：CNS, central nervous system; CS, corticosteroids; CTX, cyclophosphamide; F, female; IVIg, intravenous immunoglobulin; M, male; MTX, methotrexate; m, month; N, normal; N/A, data unavailable; y, year.

A literature search was conducted in PubMed up to December 2025 using the keywords “linear morphea en coup de sabre,” “central nervous system,” and “treatment.” The inclusion criteria comprised pediatric cases (age at diagnosis <18 years) with CNS involvement, detailed treatment records, and available outcome follow-up. The exclusion criteria for cases were as follows: lack of specified dosage and treatment duration in the therapeutic regimen, and insufficient data on post-treatment efficacy follow-up. Fifteen cases meeting these criteria were identified.
